# Methodological considerations in long-term diabetes mortality studies

**DOI:** 10.20945/2359-4292-2026-0096

**Published:** 2026-08-23

**Authors:** Chris Munayco Carhuamaca

**Affiliations:** 1 Universidad Ricardo Palma, Faculty of Human Medicine, Lima, Peru

Dear Editor,

I read with great interest the article entitled “Death cause and age at death among individuals with diabetes: a 40-year retrospective Brazilian study” published by Lucas Casagrande Passoni Lopes and cols. (^1^). The temporal breadth of the analysis spanning four decades is noteworthy; furthermore, the use of Cox regression models provides valuable information regarding how causes of death among individuals with diabetes have evolved over time. However, I consider it pertinent to highlight some methodological considerations that may influence the interpretation of the results.

An important aspect to consider is the management of the temporal dimension. Although the study incorporated decade of death as a variable, it would be pertinent to consider the possible changes that occurred throughout the four decades evaluated in diabetes management, treatment availability, and modifications in death registration systems. Patients who lived longer had access to therapeutic advances and improved diagnostic systems, which could influence their prognosis. The omission of these changes could introduce calendar-time bias and distort estimates in longitudinal studies (^2^).

At a global level, challenges in death certification and registration still persist and may affect the accuracy of epidemiological data (^3^). In this context, the study relies on death certificates as the primary source for determining the etiological classification of mortality. Although this source is widely used, it may generate classification bias, particularly in chronic diseases such as diabetes mellitus.

It is particularly relevant that authors from the same research group previously reported a high rate of underreporting of diabetes mellitus as a cause of death, reaching up to 64.41%. This situation was observed mainly among patients with type 2 diabetes mellitus and in men (^4^). This finding is especially important because it reveals a systematic information bias in the death certificates used as a data source. Since these same subgroups showed significant associations in the present study, it is possible that the results were influenced by inaccuracies in the way the etiology of death was recorded. Additionally, the accuracy of a death certificate depends largely on the certifying physician’s judgment and interpretation of the causal chain, which becomes particularly complex when patients present with multiple comorbidities (^5^).

Taken together, these points suggest that future studies could consider analyzing data according to time periods, exploring the impact of potential errors in the registration of causes of death, and incorporating clinical variables that may help improve the interpretation of the results.

## Data Availability

the authors are willing to share all data related to the study upon reasonable request.
